# A Three-Stage Algorithm to Make Toxicologically Relevant Activity Calls from Quantitative High Throughput Screening Data

**DOI:** 10.1289/ehp.1104688

**Published:** 2012-05-10

**Authors:** Keith R Shockley

**Affiliations:** Biostatistics Branch, National Institute of Environmental Health Sciences, National Institutes of Health, Department of Health and Human Services, Research Triangle Park, North Carolina, USA

**Keywords:** activity calls, concentration–response, Hill equation, quantitative high throughput screening, Tox21

## Abstract

Background: The ability of a substance to induce a toxicological response is better understood by analyzing the response profile over a broad range of concentrations than at a single concentration. *In vitro* quantitative high throughput screening (qHTS) assays are multiple-concentration experiments with an important role in the National Toxicology Program’s (NTP) efforts to advance toxicology from a predominantly observational science at the level of disease-specific models to a more predictive science based on broad inclusion of biological observations.

Objective: We developed a systematic approach to classify substances from large-scale concentration–​response data into statistically supported, toxicologically relevant activity categories.

Methods: The first stage of the approach finds active substances with robust concentration–response profiles within the tested concentration range. The second stage finds substances with activity at the lowest tested concentration not captured in the first stage. The third and final stage separates statistically significant (but not robustly statistically significant) profiles from responses that lack statistically compelling support (i.e., “inactives”). The performance of the proposed algorithm was evaluated with simulated qHTS data sets.

Results: The proposed approach performed well for 14-point-concentration–response curves with typical levels of residual error (σ ≤ 25%) or when maximal response (|*RMAX*|) was > 25% of the positive control response. The approach also worked well in most cases for smaller sample sizes when |*RMAX*| ≥ 50%, even with as few as four data points.

Conclusions: The three-stage classification algorithm performed better than one-stage classification approaches based on overall *F*-tests, *t*-tests, or linear regression.

The goals of the Tox21 collaboration are to prioritize chemicals for *in vivo* testing, identify mechanisms of toxicity, and predict adverse responses to environmental chemicals in humans (Collins et al. 2008; Shukla et al. 2010). Low throughput animal and tissue models are yielding ground to high throughput screening (HTS) methods that enable the simultaneous assessment of large numbers of compounds. For applications of HTS in traditional drug discovery, assays are usually conducted at a single test concentration (e.g., 10 μM) to find compounds with strong pharmacological activity while reducing the risk for false positives. This strategy is not as relevant for toxicological research and toxicity testing, which also seeks to find compounds with weak activity while reducing the risk for false negatives. However, quantitative high throughput screening (qHTS) provides an opportunity to meet Tox21 objectives, holding the potential for wide chemical coverage and reduced cost of testing on a per-substance basis. Moreover, the ability of a substance to induce a toxicological response is better understood by analyzing the response profile over a broad concentration range than by evaluating effects at one or a few concentrations.

The Tox21 collaboration began formally in 2008 with Phase I (Proof of Concept) consisting of qHTS studies conducted at the National Institutes of Health Chemical Genomics Center (NCGC) in 1,536-well–format and mid-throughput studies conducted in support of the U.S. Environmental Protection Agency’s (EPA) ToxCast™ program. In conjunction with Tox21 Phase I, the NTP and U.S. EPA have produced an extensive set of concentration–response data on some 2,800 substances screened at the NCGC in > 70 qHTS assays and on 320 substances tested across > 500 *in vitro* and lower organism *in vivo* assays by various contract and government laboratories. In Tox21 Phase II, qHTS data will soon be produced for a library containing approximately 10,000 compounds. Analyses of Phase I data indicate reproducible levels of compound behavior that match previously known toxicological responses (Huang et al. 2008). These experiments are typically analyzed using a heuristics-based curve classification algorithm that does not use uncertainty in model fits to make activity calls (Inglese et al. 2006). However, classification of chemical activity has also been based on clustering by pattern dissimilarity (Zhang et al. 2009), a heuristics approach incorporating curve fit *p*-values (Huang et al. 2011), testing for significance of response using mathematical models (Parham et al. 2009), or a preliminary test estimation (PTE) procedure robust to variance structure (S. Peddada, personal communication).

Because of the potential for complex concentration–response behavior, toxicological evaluation has traditionally been based on manual scrutiny of concentration–response (or dose–response) data. But the large data volume surrounding qHTS renders manual inspection of individual profiles restrictively laborious, subjective, and prone to human error. Indeed, the human eye cannot consistently discriminate calls based on small (but statistically relevant) trends or differences, and conventional curve fit diagnostics are not feasible when considering the large number of compounds used within qHTS studies. Heuristics approaches to screen qHTS data sets may identify candidates with positive activity, but such methods are not based on the principles of statistical hypothesis testing. On the other hand, statistical assessments based on fits to a nonlinear function may not capture important responses occurring outside of the specified model framework. For instance, a maximal response at the lowest tested concentration will not be adequately explained by fitting the conventional Hill equation (Hill 1910). Given these considerations, there is currently no suitable approach for making statistically rigorous activity calls in an automated manner for the massive amount of data emerging from large-scale toxicity testing within the NTP and Tox21 qHTS efforts. In addition, the operating characteristics of the limited number of activity call algorithms published to date have not yet been systematically explored in the published literature.

To meet this need, we propose a three-stage framework based on formal statistical testing of toxicologically relevant hypotheses. Although much of the data generated to date has been produced from unreplicated designs, this approach can accommodate various levels of replication and provides a consistent platform for making activity calls. In the first stage of the algorithm, compounds with a robust concentration–response relationship are identified by comparing the best fit to a nonlinear model with a horizontal line (no concentration–response) and classified as “active.” Compounds not detected as “active” in the first stage are tested for activity at the lowest tested concentration in the second stage. Finally, compounds with a statistically less robust concentration–response are classified as “inconclusive” and distinguished from “inactive” calls in the third stage. Receiver operating characteristic (ROC) curves of simulated qHTS data are used to assess the overall ability of the algorithm to detect active compounds under toxicologically relevant conditions produced in simulated data sets.

## Methods

*Development of the algorithm.* Our approach assumes that the toxicological importance of a response profile generated in qHTS applications should be determined by a robust framework to impartially classify tested substances and limit the return of false negatives. A set of simple decision rules are used to make consistent activity calls from the wealth of complex response patterns resulting from high throughput chemical profiling. These decision rules are formalized with statistical procedures and automated through a systematic computational workflow. Substances classified as “actives” have response values exceeding the assay detection limit (see below) and may fall into one of two different categories: *a*) compounds with concentration–response curves within the tested concentration range, and *b*) compounds eliciting maximal responses at the lowest tested concentration. The first category of substances can be subdivided into two subgroups: *a*) compounds producing statistically and toxicologically robust concentration–response trends supported by multiple data points in different regions of each profile (see “Stage 1: Test for robust concentration–response,” below), and *b*) compounds that may fit sigmoidal curves better than flat lines in a mathematical or statistical sense but are comparatively nonrobust by toxicological standards (e.g., curves with only one data point exceeding the detection limit). Substances underlying such nonrobust concentration–response profiles are labeled “inconclusive.” “Inconclusive” calls may arise due to low levels of replication, variability in assay performance, or confounding of factors in nonrandomized designs (e.g., experimental drift of scanning machines).

**Figure 1:**
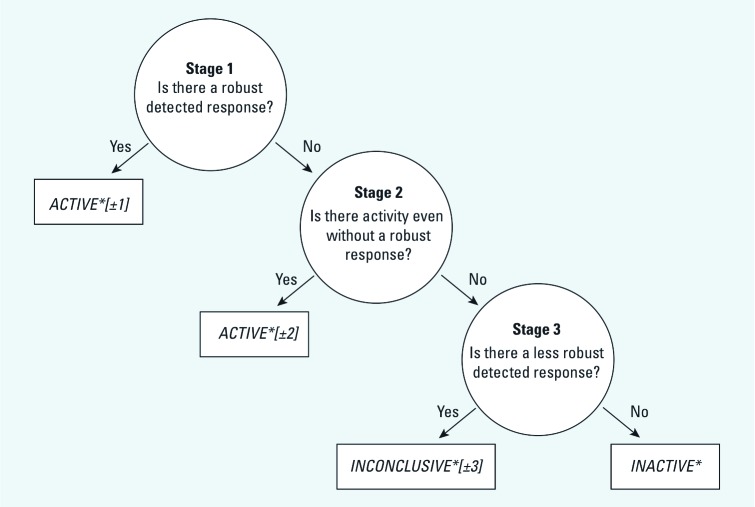
Three-stage algorithm used to classify the activity of a substance from normalized qHTS data. The tree is defined by stages (circles), where the result of each stage determines the next stage to apply. The process continues until the path terminates in a call (rectangles). The number in the brackets designates the direction of the assay as described in the text (“+” refers to activation; “–“ refers to inhibition).

A three-stage algorithm (Figure 1) is proposed to classify each substance in a tested chemical library as “active,” “inactive,” or “inconclusive” (Table 1). There are two types of actives: *a*) ACTIVE*[±1] substances describe robust concentration–response curves and *b*) ACTIVE*[±2] agents are putatively active ≤ the lowest tested concentration. Less robust responses are assigned INCONCLUSIVE*[±3] and substances with no discernable activity within the tested concentration range are classified as INACTIVE*. Numbers inside brackets refer to the stage where the call was made (i.e., *STAGE 1*, *STAGE 2*, or *STAGE 3* in Figure 1). The “+” or “–” sign inside each bracket corresponds to the direction of the response. Accordingly, ACTIVE*[1] and INCONCLUSIVE*[3] describe response curves in which the response signal tends to increase with increasing concentration (activators), whereas ACTIVE*[–1] and INCONCLUSIVE*[–3] describe response curves in which the response signal tends to decrease with increasing concentration (inhibitors). Calls labeled ACTIVE*[2] (or ACTIVE*[–2]) refer to substances with mean responses significantly greater (or lower) than the detection limit of the assay.

**Table 1:** Criteria for classification algorithm.

Stage/condition		Activity call
Stage 1			
	(1) MAX(Ria) > positive DetLimb		ACTIVE*[1] (activator)
	(2) H0: Ri = Σ Ri/nc is rejected for F-test (NLSd fit) and H0: Ri = Σ wieRi/n is rejected for F-test (WNLSf fit)		
	(3) RMAXg > R0h (NLS fit) and RMAX > R0 (WNLS fit)		
	(1) MIN(Ri) < negative DetLim		ACTIVE*[–1] (inhibitor)
	(2) H0: Ri = Σ Ri/n is rejected (NLS fit) and H0: Ri = Σ wiRi/n is rejected (WNLS fit)		
	(3) RMAX < R0 (NLS fit) and RMAX < R0 (WNLS fit)		
Stage 2			
	(1) Not active in Stage 1		ACTIVE*[2] (potent activator)
	(2) H0: Ri ≤ DetLim is rejected using weighted t-test		
	(1) Not active in Stage 1		ACTIVE*[–2] (potent inhibitor)
	(2) H0: Ri ≥ DetLim is rejected using weighted t-test		
Stage 3			
	(1) Not active in Stage 1 or Stage 2		INCONCLUSIVE*[3] (putative activator)
	(2) MAX(Ri) > positive DetLim		
	(3) H0: Ri = Σ Ri/n is rejected for F-test (NLS fit) and (4.a) or H0: Ri = Σ wiRi/n is rejected for F-test (WNLS fit) and (4.b)		
	(4.a) RMAX > R0 (NLS fit)		
	(4.b) RMAX > R0 (WNLS fit)		
	(1) Not active in Stage 1 or Stage 2		INCONCLUSIVE*[–3] (putative inhibitor)
	(2) MIN(Ri) < negative DetLim		
	(3) H0: Ri = Σ Ri/n is rejected (NLS fit) and (4.a) or H0: Ri = Σ wiRi/n is rejected (WNLS fit) and (4.b)		
	(4.a) RMAX < R0 (NLS fit)		
	(4.b) RMAX < R0 (WNLS fit)		
	(1) Not active in Stage 1 or Stage 2 or Stage 3		INACTIVE*
aRi, response at concentration i. bDetLim, magnitude of the detection limit in a typical qHTS assay is generally 25–30% of the measured positive control response. cn, total number of concentrations tested. dNLS, nonlinear least squares regression. ewi, weight for Ri. fWNLS, weighted nonlinear least squares regression. gRMAX, maximal activity from the Hill Equation. hR0, baseline activity from the Hill Equation. [For more detail, see Supplemental Material, pp. 3–4 (http://dx.doi.org/10.1289/ehp.1104688)].			

Detection limits define a response range in which the normalized signal can be reliably measured within a given experiment, and are usually set to 3 SD above or below the normalized signal intensities observed in negative control plates. A detection limit of 25–30% of the positive control is typical within Tox21 efforts. The positive detection limit for activator assays is found by adding the assay noise level to the control response; the negative detection limit for inhibitor assays is calculated by subtracting the assay noise level from the control response.

**Figure 2:**
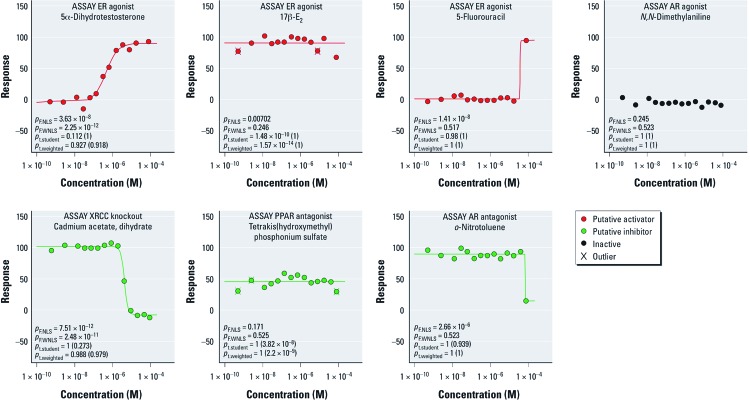
Example response profiles from experimental data obtained within Tox21 qHTS studies. *p*-Values shown are from the overall *F*-test using the nonlinear least squares approach (*p*_F.NLS_), the overall *F*-test using the weighted nonlinear least squares approach (*p*_F.WLS_), Student’s *t*-test comparing the mean response to 25% response followed by comparison to –25% response in parentheses (*p*_t.student_), and a weighted *t*-test comparing the mean response to 25% response followed by comparison to –25% response in parentheses (*p*_t.weighted_). Activity calls resulting from the proposed algorithm are indicated on the figure.

Example concentration–response profiles and their activity calls from qHTS data generated with the NTP compound library used in Tox21 Phase I are shown in Figure 2. More extensive data can be found in NTP’s Chemical Effects in Biological Systems database (Waters et al. 2008).

The following form of the Hill equation model is used here:


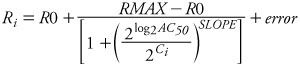
[1]

where *R_i_* is the response value for concentration *i*, *R*0 is the baseline activity (lower asymptote of the sigmoidal curve for activators, upper asymptote of the sigmoidal curve for inhibitors), *RMAX* is the maximal activity (upper asymptote for activators, lower asymptote for inhibitors), and |*RMAX* – *R*0| defines the maximal response compared to baseline activity for activators and inhibitors. In Equation 1, *C_i_* refers to the test concentration of each response, *AC_50_* is the concentration yielding 50% of the maximal response produced by the tested compound (i.e., EC_50_ for activators, IC_50_ for inhibitors), *SLOPE* determines the shape of the curve and *error* is the residual error of the model. [For technical details describing curve fitting, see Supplemental Material, p. 3 (http://dx.doi.org/10.1289/ehp.1104688)].

*Stage 1: Test for robust concentration–response.* The objective of the first stage is to find chemicals with a robust dose–response relationship within the tested concentration range. To satisfy the robust criterion, response profiles should exhibit statistical significance in both unweighted nonlinear least squares (NLS) and weighted nonlinear least squares (WNLS) regression approaches [see Supplemental Material, p. 3 (http://dx.doi.org/10.1289/ehp.1104688)]. The NLS approach weights all data points equally and, consequently, may not discriminate adequately between a profile with data along two clearly defined asymptotes and a less robust profile in which one asymptote is supported by a single point (Figure 2). In contrast, the WNLS criterion weights each response point *i* from *n* concentrations (*R_i_*, *i* = 1, …, *n*), so that more influence is given to neighboring data points with similar response levels than neighboring data points with very different responses. An active compound from Stage 1 (i.e., ACTIVE*[±1]) will

Have a maximum response greater than positive detection limit (for activators) or a minimum response less than the negative detection limit (for inhibitors).
Fit the four-parameter Hill model better than a horizontal line using both NLS and WNLS regression at a selected significance level.
Show increasing response (*RMAX > R*0 for ACTIVE*[1] calls) or decreasing response (*RMAX < R*0 for ACTIVE*[–1] calls) with increasing concentration.

*Stage 2: Test for activity at lowest tested concentration.* In the second stage, compounds not detected as active in the first stage are evaluated by comparing the distribution of measured responses to the detection limit of the assay. Compounds with activity at the lowest tested concentration are considered to be relatively potent since their *AC*_50_ values are presumably less than (or equal to) the lowest tested concentration. To find these “potent responders,” tests for mean response greater than the detection limit of the assay are performed using weighted *t*-tests with the same weighting scheme used for WNLS [see Supplemental Material, p. 4 (http://dx.doi.org/10.1289/ehp.1104688)]. The weighted *t*-test ensures that outlier responses are not given unwarranted weight in statistical assessment. A compound active in this stage will have activity at the lowest tested concentration that is greater than the positive assay detection limit (ACTIVE*[2]) or decreased activity at the lowest tested concentration that is lower than the negative assay detection limit (ACTIVE*[–2]).

*Stage 3: Test for nonrobust concentration–response.* In the third stage, compounds not detected as active in the first or second stage are evaluated. Compounds with significant fits to the Hill model using either NLS or WNLS, but not both, are classified as INCONCLUSIVE*[±3]. For example, profiles found in Stage 3 may be incomplete curves in which the curve fit is driven by a single data point at a high test concentration (Figure 2). An inconclusive compound will

Have a maximum response greater than positive detection limit (for activators) or a minimum response less than the negative detection limit (for inhibitors).
Fit the four-parameter Hill model better than a horizontal line (no response) in either the unweighted (NLS) or weighted (WNLS) curve fit.
Show increasing response (*RMAX* > *R*0 for INCONCLUSIVE*[3]) or decreasing response (*RMAX < R*0 for INCONCLUSIVE*[–3]) with increasing concentration.

Compounds that are not classified as active or inconclusive in Stage 1, Stage 2, or Stage 3 are putatively assigned inactive.

***Simulation study.*** Activators (*RMAX* > *R*0) and inhibitors (*RMAX* < *R*0) simulated from Equation 1 will produce fully symmetric profiles and yield identical performance metrics for equivalent *R*0, |*RMAX* – *R*0|, *AC_50_* and *SLOPE*. Therefore, simulations were conducted only for activators. A total of 10,000 hypothetical substances were simulated for each concentration–response data set with *R*0 = 0%, where each data set included 2,000 simulated actives (|*RMAX*| ≥ 25% of positive control activity) and 8,000 simulated inactives (*RMAX* = 0%). Three different simulation settings were explored (see Table 2). Case 1 explored the performance of the algorithm under different error structures. Case 2 assessed the effect of the *SLOPE* parameter when residual error was set to a value typical of qHTS data [σ = 25%; see Supplemental Material, Table S1 (http://dx.doi.org/10.1289/ehp.1104688)]. Case 3 examined the performance of the algorithm when various numbers of data points (1, 3, 5, 7, or 10) were removed to produce a smaller effective sample size *n*. The R package “drc” (Ritz and Streibig 2005) was used to fit all concentration–response models, and outlier detection followed a two-step algorithm that was combined with curve fitting (Wang et al. 2010). (For more information about the simulation see Supplemental Material, pp. 4–5)

**Table 2:** Parameter values used in the simulations.

Simulation feature	Case 1^a^	Case 2	Case 3^a^
True AC50 values	(10–3, 10–1, 10)	(10–3, 10–1, 10)	(10–3, 10–1, 10)
True |RMAX| values	(25, 50, 100)	(25, 50, 100)	(25, 50, 100)
True R0 values	0	0	0
True SLOPE values	1	(0.01, 0.1, 0.5, 1, 2, 10, 100)	1
Number of parameter configurations	9b	63	9b
Residual ERROR structures (σ)c	(5%, 10%, 25%, 50%, 100%, f(Ci))	25%	25%
No. of data points (n)	14	14	(4, 7, 9, 11, 13)
aA more extensive parameter space of 49 parameter configurations was used to generate contour plots for Case 1 (Figure 3), where AC50 values (μM) were set to (10–4, 10–3, 10–2, 10–1, 1, 10, 100) and |RMAX| values (percentage of positive control) were set to (10, 25, 50, 75, 100, 125, 150). bThe 49 parameter configurations from footnote a, above, define a more extensive parameter space that is used to generate contour plots. cResidual error values were modeled as ε ~ N(0, σi2) for σi = (5%, 10%, 25%, 50%, 100%, and f(Ci)), where σi is expressed as percent of positive control activity at concentration i and f(Ci) = 9.7355 + 0.1146 × Ci. [For more detail, see Supplemental Material, Equation 1 (http://dx.doi.org/10.1289/ehp.1104688).]			

Type I error rates were estimated for null hypothesis cases (*RMAX* = 0%) by evaluating the empirical proportions of trials in which the algorithm assigned a simulated null hypothesis as active (ACTIVE*[±1] or ACTIVE*[±2]). For computational purposes, INCONCLUSIVE*[±3] calls were treated as inactive. Sensitivities were estimated by evaluating the empirical proportions of true active cases (|*RMAX*| ≥ 25%) assigned as active (ACTIVE*[±1] or ACTIVE*[±2]). In all cases, the significance level (α) for statistical testing was set to 0.05.

The area under receiver operating characteristic (ROC) curves was used as the primary statistic to assess performance. ROC graphs describe the relationship between sensitivity (true positive rate or power) and 1-specificity (false positive rate or type I error rate) of a classification method and are not influenced by skewed class distribution or unequal classification error costs (Fawcett 2006). The area under the curve (AUC) of each ROC graph was calculated using the trapz() function in the R package “caTools” (Tuszynski 2009). AUC ranges from 0.0 to 1.0 and provides a probability describing how well the algorithm can correctly classify true actives and true inactives based on the known parameter values used to simulate the data. Random performance is indicated by AUC = 0.5. Here, AUC = 0.75 is chosen to indicate good performance, whereas AUC = 0.9 indicates excellent performance.

## Results

*Analysis of androgen receptor agonist assay data.* Chemical genomics profiling data from a previously published androgen agonist assay was obtained for the 1,408 compounds in the NTP Tox21 compound collection (Huang et al. 2011). Compounds in that study were dissolved in dimethyl sulfoxide and exposed to 14 concentrations ranging from 4.90 × 10^–4^ μM to 76.63 μM. For *p* < 0.05, the three-stage algorithm proposed here classified 82 compounds as active (26 activators and 58 inhibitors), 100 compounds as inconclusive (55 activators and 44 inhibitors), and the remaining 1,225 compounds as inactive. These calls were compared to activity calls generated by a curve class procedure (Huang et al. 2011) and the Parham method (Parham et al. 2009) and results obtained from single-stage tests, including *F*-tests based on NLS or WNLS curve fits, robust linear regression, Student’s *t*-tests and weighted *t*-tests (Table 3). There was substantial overlap and notable differences between these outcomes even though all approaches used the same statistical significance threshold (*p* < 0.05). Of the 26 activator hits identified by the three-stage algorithm, the curve class method placed 15 in curve class 1 (full sigmoidal response profiles), 8 in curve class 2 (partial response profiles with one asymptote), and 1 compound each into curve classes 3 (single point activity), 4 (inactive) and 5 (undefined). The Parham method shared 19 of the 26 three-stage actives, with 2 inconclusive activators, 1 inconclusive inhibitor, and 4 inactives. The NLS and WLS methods each contained the same 26 compounds in common with the three-stage approach, but the robust linear regression approach had only 21 of the 26 actives in common. Calls based on Student’s *t*-test and the weighted *t*-test shared 11 and 7 compounds, respectively, in common with the three-stage approach. The full comparison between approaches is presented in Table 3.

**Table 3:** Comparing activity calls from the three-stage approach to other methods for an androgen receptor agonist qHTS assay.^a^

Activity call strategy	ACTIVE*[1]	ACTIVE*[–1]	ACTIVE*[2]b	ACTIVE*[–2]	INCONCL*[3]	INCONCL*[–3]	INACTIVE*
Three-stage approach		26		56		0		2		55		44		1225
Revised NCGC curve classc														
1.1 (–1.1)		8 (0)		0 (0)		—		0 (0)		0 (0)		0 (0)		0 (0)
1.2 (–1.2)		2 (0)		0 (11)		—		0 (0)		0 (0)		0 (0)		0 (0)
1.3 (–1.3)		2 (0)		0 (0)		—		0 (0)		0 (0)		0 (0)		0 (0)
1.4 (–1.4)		3 (0)		0 (6)		—		0 (0)		0 (0)		0 (1)		0 (4)
2.1 (–2.1)		3 (0)		0 (0)		—		0 (0)		3 (0)		0 (0)		0 (0)
2.2 (–2.2)		1 (0)		0 (15)		—		0 (0)		3 (0)		0 (9)		0 (2)
2.3 (–2.3)		1 (0)		0 (0)		—		0 (0)		2 (0)		0 (0)		0 (0)
2.4 (–2.4)		3 (0)		0 (20)		—		0 (0)		12 (0)		0 (16)		2 (19)
3 (–3)		1 (0)		0 (3)		—		0 (0)		19 (0)		0 (15)		5 (7)
4		1		1		—		2		11		1		1186
5		1		0		—		0		5		2		0
Parham methodd														
Active INCR (DECR)		19 (0)		0 (0)		—		1 (0)		11 (0)		0 (0)		4 (0)
Inconclusive INCR (DECR)		2 (1)		2 (30)		—		0 (0)		20 (1)		1 (14)		36 (28)
Inactive		4		24		—		1		23		29		1157
Actives from other approaches														
NLS F-test INCR (DECR)e		26 (0)		0 (56)		—		1 (1)		53 (0)		0 (43)		86 (270)
WNLS F-test INCR (DECR)f		26 (0)		0 (56)		—		0 (0)		2 (7)		1 (1)		64 (402)
Robust linear regression m > 0 (m < 0)g		21 (0)		0 (49)		—		0 (1)		11 (0)		0 (23)		1 (2)
Student’s t-test μ > 25% (μ < –25%)		11 (0)		0 (0)		—		0 (2)		0 (0)		0 (0)		0 (0)
Weighted t-test μ > 25% (μ < –25%)		7 (0)		0 (1)		—		0 (2)		0 (0)		0 (0)		0 (0)
aShows the number of predicted activators (or inhibitors, in parentheses) for each activity call strategy that are shared with the three-stage approach. bMissing data because there are no ACTIVE*[2] calls. cSee Huang et al. (2011). dSee Parham et al. (2009). eNonlinear least squares F-test and fweighted nonlinear least squares with RMAX > R0 (activators) or RMAX < R0 (inhibitors). gCalculated using rlm() function in R package “MASS” (Venables and Ripley 2002).														

*Overview of simulation studies.* The performance of the algorithm was investigated for all 171 simulated qHTS data sets by examining combinations of *AC_50_* (three levels), |*RMAX*| (three levels), and *R*0 (one level) for three different cases (Table 2). Case 1 varied the error structure, Case 2 varied the Hill slope, and Case 3 varied the number of available data points. An AUC corresponding to each ROC curve was calculated for each parameter configuration, except when σ = 5% (9 data sets) since no false positives were returned under this condition. Resulting AUCs from the remaining 162 data sets were compared with the proposed algorithm versus overall *F*-tests comparing the fit to the Hill model and a straight line (NLS or WNLS), *t*-tests (Student’s *t*-tests or weighted *t*-tests) and robust linear regression as shown in Supplemental Material, Figure S1 (http://dx.doi.org/10.1289/ehp.1104688). In general, performance was not good (AUC ≤ 0.75) when |*RMAX*| = 25%, but the proposed algorithm showed similar or improved performance compared to overall *F*-tests in almost every scenario, and performed considerably better than overall *F*-tests for *AC_50_* = 0.001 μM. The proposed method usually outperformed *t*-tests when *AC_50_* = 10 μM (fewer data points with detectable responses), but did not perform as well as *t*-tests in some instances when *AC_50_* < 10 μM (increased number of detectable responses). The proposed approach outperformed robust linear regression in almost every scenario. Compared to the proposed method, *t*-tests generally had smaller type I error rates (see Supplemental Material, Figure S2), but *t*-tests also had noticeably reduced power when *AC_50_* > 0.001 μM (see Supplemental Material, Figure S3).

**Figure 3:**
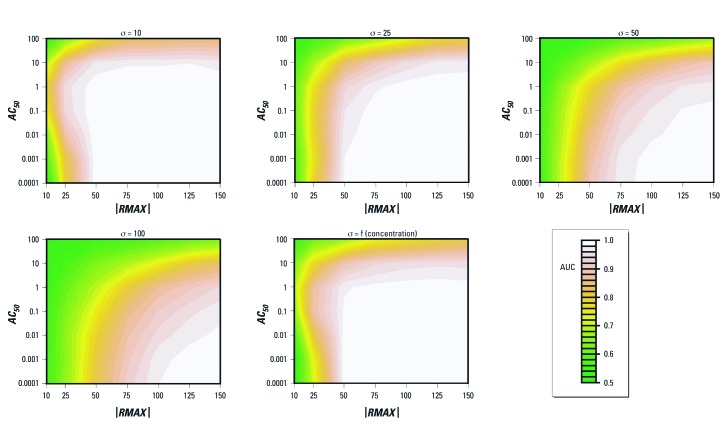
Contour plots to evaluate classification performance of proposed approach to make activity calls from 14-point concentration–response curves. The plots summarize the performance characteristics of the proposed classification algorithm based on AUC of the ROC curve generated from a broad parameter space of |*RMAX*| and *AC_50_* under different residual error scenarios. Regions of each plot with AUC ≥ 0.75 indicate moderately good performance, and regions with AUC > 0.9 represent excellent performance. The significance level for statistical tests is 0.05.

*Case 1: 14-point-concentration–response curves.* A total of 54 simulated qHTS data sets were used to evaluate the proposed algorithm for nine configurations involving changes in *AC_50_* (three levels) and |*RMAX*| (three levels) for *R*0 = 0 and *SLOPE* = 1 under six different residual error structures (Table 2). Residual errors were modeled as ε ~ N(0, σ_i_^2^) for σ_i_ = (5%, 10%, 25%, 50%, 100%, and f(*C_i_*)), where σ_i_ is expressed as percent of positive control activity at concentration *i* and f(*C_i_*) = 9.7355 + 0.1146 × *C_i_*. The function f(*C_i_*) is based on the best fit line between σ_i_ and concentration derived from qHTS data generated from human nuclear receptor agonist-mode assays [Huang et al. 2011; see also Supplemental Material, Table S1 (http://dx.doi.org/10.1289/ehp.1104688)]. Table 4 summarizes the operating characteristics of the proposed approach for Case 1. Type I (false positive) error rates do not exceed 0.05 for true inactives when σ_i_ = (5%, 10%, 25%, f(*C_i_*)), remained close to 0.05 for σ_i_ = 50%, and consistently exceeded 0.05 when σ_i_ = 100% (see also Supplemental Material, Figure S2). Notably, type I error rates increase with increasing residual error, with no false positives at σ_i_ = 5%. For known actives, the proposed approach exhibits greater power with increasing |*RMAX*|. The power decreases with increasing residual error and is almost always above 80% when |*RMAX*| = 100% in constant error (σ_i_ = 25%) and heteroscedastic error (σ_i_ = f(*C_i_*)) scenarios (see Supplemental Material, Figure S3). As shown in Table 4, the proposed algorithm performed well (AUC ≥ 0.75) for scenarios with typical levels of residual error (σ ≤ 25% in most cases in Supplemental Material, Table S1), and with even better performance (AUC ≥ 0.9) for |*RMAX*| > 25%. Table 4 also illustrates that an increasing proportion of activity calls are ACTIVE*[2] (rather than ACTIVE*[1]) with increasing residual error. Figure 3 summarizes the performance of the proposed approach using contour plots and indicates that AUC > 0.75 for all levels of *AC_50_* within the tested range when |*RMAX*| > 25%. Performance diminished with increasing residual error, and for σ = 100% the approach was only better than random prediction for large maximal responses (|*RMAX*| > 75%) and lower potencies (*AC_50_* < 1 μM).

**Table 4:** Case 1 error rates and power of proposed method for different residual error structures.^a^

True AC_50_	True |RMAX|	Type I error rate											Power										
		5%^b^	10%	25%	50%	100%	f(C_i_)	5%^b^	10%	25%	50%	100%	f(C_i_)
0.001		25		0.000		0.001 (100)		0.021 (85.8)		0.054 (37.0)		0.118 (17.4)		0.007 (100)		0.314** (72.1)		0.228* (40.4)		0.206* (18.2)		0.229 (11.4)		0.237 (11.8)		0.201 (40.9)
0.001		50		0.000		0.001 (100)		0.020 (87.9)		0.059 (38.7)		0.116 (18.4)		0.006 (100)		1.000** (26.6)		0.991** (22.9)		0.855** (7.7)		0.598** (7.3)		0.406 (6.9)		0.987** (20.3)
0.001		100		0.000		0.001 (100)		0.024 (86.8)		0.054 (42.1)		0.124 (20.7)		0.010 (100)		1.000** (19.1)		1.000** (27.0)		0.999** (15.3)		0.963** (8.2)		0.773* (5.8)		1.000** (26.8)
																										
0.1		25		0.000		0.001 (100)		0.023 (87.3)		0.060 (41.3)		0.127 (19.6)		0.008 (100)		0.966** (99.9)		0.664** (99.5)		0.197* (73.1)		0.188 (34.6)		0.206 (18.0)		0.576* (99.3)
0.1		50		0.000		0.001 (100)		0.020 (87.4)		0.065 (40.1)		0.122 (17.4)		0.010 (100)		1.000** (99.5)		0.996** (98.1)		0.684** (71.2)		0.403* (37.2)		0.324 (21.3)		0.990** (98.7)
0.1		100		0.000		0.001 (100)		0.024 (84.9)		0.062 (37.3)		0.119 (16.2)		0.008 (100)		1.000** (99.6)		0.999** (99.4)		0.994** (94.4)		0.850** (55.0)		0.582* (27.1)		1.000** (99.6)
																										
10		25		0.000		0.001 (100)		0.022 (88.3)		0.059 (40.7)		0.127 (17.9)		0.007 (100)		0.366** (100)		0.332* (100)		0.100 (93.5)		0.111 (47.7)		0.154 (26.3)		0.275* (100)
10		50		0.000		0.0004 (100)		0.022 (89.0)		0.060 (35.9)		0.118 (18.8)		0.009 (100)		0.952** (100)		0.896** (99.9)		0.328* (89.9)		0.194 (51.3)		0.207 (24.5)		0.773* (99.9)
10		100		0.000		0.001 (100)		0.019 (92.0)		0.057 (37.4)		0.123 (20.2)		0.010 (100)		0.948** (100)		0.955** (100)		0.791** (97.3)		0.440* (66.7)		0.315 (30.3)		0.916** (100)
aType I error rates and power are shown as a fraction ranging from 0 to 1, with the percentage of ACTIVE*[1] actives out of the total actives (equal to ACTIVE*[1]/(ACTIVE*[1] + ACTIVE*[2]) × 100%) indicated in parentheses. bFor 5% residual error, there were no false positives in the simulation. *AUC ≥ 0.75. **AUC ≥ 0.9.																										

**Figure 4:**
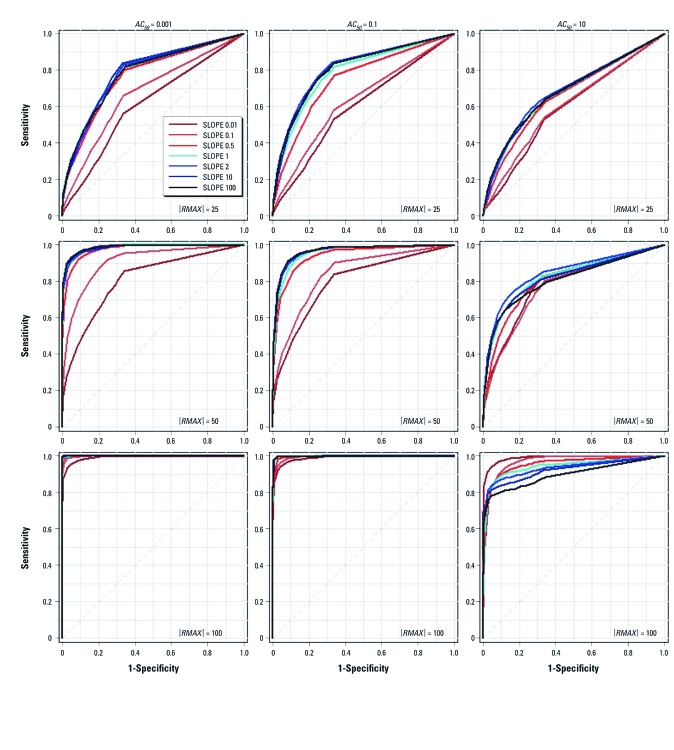
Case 2 ROC curves for different parameter configurations for σ = 25% error. Sensitivity versus (1 – Specificity) are plotted for 63 different parameter configurations of *AC_50_* (0.001, 0.1, 10 μM), |*RMAX*| (25%, 50%, 100%), and *SLOPE* (0.01, 0.1, 0.5, 1, 2, 10, 100) for *R0* = 0. The diagonal line indicates random performance. The significance level for statistical tests is 0.05.

*Case 2: Evaluating the SLOPE parameter.* Combinations of *AC_50_* (three levels), |*RMAX*| (three levels), and *SLOPE* (seven levels) were used to investigate the performance of 63 parameter configurations for a range of *SLOPE* parameter settings (Table 2). As shown in Figure 4, performance was similar for most *SLOPE* settings, where *SLOPE* varied from *SLOPE* = 10^–4^ to *SLOPE* = 100 and |*RMAX*| took one of three values (25%, 50%, 100%). At |*RMAX*| = 25%, the proposed algorithm performed poorly for every parameter configuration, while at |*RMAX*| = 100%, the proposed approach performed similarly well for every *SLOPE* parameter value examined. When |*RMAX*| = 50%, most parameter configurations yielded similar performance, except when *SLOPE* ≤ 0.5.

*Case 3: Effects of sample size.* A defined number of data points (0, 1, 3, 5, 7, or 10) were randomly removed within each substance in each 14-point data curve in order to evaluate the operating characteristics of the proposed algorithm for different sample sizes *n* (Tables 2 and 5). While type I error rates generally increased with *n*, they were < 0.03 in every case examined here. Power to detect actives increased with increasing *n*. The performance of the proposed approach was good in most cases (AUC ≥ 0.75) with |*RMAX*| set to 50% or 100% and *AC_50_* set to 0.001 μM or 0.1 μM, even with as few as four data points. However, the power was greatly reduced when |*RMAX*| = 25%. As shown in Table 5, an increasing proportion of activity calls are ACTIVE*[1] (rather than ACTIVE*[2]) with increasing sample size.

**Table 5:** Case 3 error rates and power of proposed method at 25% residual error for different sample sizes (*n*).^a^

True AC_50_	True |RMAX|	Type I error rate											Power										
		4	7	9	11	13	14^b^	4	7	9	11	13	14^b^
0.001	25		0.004 (0.0)		0.008 (32.3)		0.014 (72.1)		0.019 (79.2)		0.019 (83.2)		0.021 (85.8)		0.076 (0.0)		0.164* (7.0)		0.175* (11.1)		0.202* (20.3)		0.203* (22.4)		0.206* (18.2)
0.001	50		0.006 (0.0)		0.008 (57.4)		0.012 (75.0)		0.017 (82.7)		0.018 (90.7)		0.020 (87.9)		0.336** (0.0)		0.687** (1.5)		0.759** (4.1)		0.821** (6.0)		0.840** (6.7)		0.855** (7.7)
0.001	100		0.005 (0.0)		0.009 (39.7)		0.012 (79.3)		0.016 (85.8)		0.020 (83.5)		0.024 (86.8)		0.682** (0.0)		0.987** (1.9)		0.994** (5.5)		0.999** (10.0)		0.998** (14.5)		0.999** (15.3)
																									
0.1	25		0.005 (0.0)		0.008 (40.3)		0.011 (72.4)		0.018 (80.7)		0.021 (84.7)		0.023 (87.3)		0.053 (0.0)		0.101 (20.9)		0.132* (45.6)		0.177* (62.7)		0.191* (71.7)		0.197* (73.1)
0.1	50		0.006 (0.0)		0.008 (39.1)		0.016 (76.2)		0.018 (84.6)		0.021 (88.8)		0.020 (87.4)		0.174* (0.0)		0.350** (18.3)		0.498** (37.0)		0.576** (53.0)		0.655** (64.8)		0.684** (71.2)
0.1	100		0.005 (0.0)		0.010 (46.8)		0.013 (77.6)		0.018 (85.7)		0.021 (88.1)		0.024 (84.9)		0.432* (0.0)		0.797** (31.2)		0.922** (63.4)		0.974** (82.6)		0.995** (92.7)		0.994** (94.4)
																									
10	25		0.005 (0.0)		0.011 (37.8)		0.013 (74.8)		0.017 (79.0)		0.021 (80.8)		0.022 (88.3)		0.015 (0.0)		0.029 (54.4)		0.058 (75.0)		0.082 (89.6)		0.107 (89.7)		0.100 (93.5)
10	50		0.004 (0.0)		0.008 (43.5)		0.013 (71.0)		0.018 (85.8)		0.023 (90.0)		0.022 (89.0)		0.021 (0.0)		0.070 (56.1)		0.158* (73.7)		0.205* (83.2)		0.276* (89.7)		0.328* (89.9)
10	100		0.004 (0.0)		0.009 (39.1)		0.014 (68.8)		0.018 (90.8)		0.021 (88.1)		0.019 (92.0)		0.060 (0.0)		0.217* (61.0)		0.417* (83.4)		0.620** (92.4)		0.761** (96.8)		0.791** (97.3)
aShown are the type I error rates and power as a fraction ranging from 0 to 1, with the percentage of ACTIVE*[1] actives out of the total actives (equal to ACTIVE*[1] / (ACTIVE*[1] + ACTIVE*[2]) × 100%) indicated in parentheses. bThe type I error rates and sensitivities from Case 1 (n = 14) are shown here for comparison. *AUC ≥ 0.75. **AUC ≥ 0.9.																									

## Discussion

Assessment of health risks posed by an environmental chemical generally proceeds through costly and time intensive studies such as the 2-year rodent bioassay. These *in vivo* assays can take several years to complete and cost millions of dollars. Yet, an estimated 30,000 unique chemicals are in wide commercial use (Judson et al. 2008; Muir and Howard 2006) and most of these substances have not been tested for adverse effects on humans or the environment. Accordingly, there is a need to prioritize chemicals for standard toxicity testing and to find alternative strategies to evaluate the large inventory of potentially harmful substances (Judson et al. 2010). qHTS holds potential to meet these objectives by augmenting the low throughput animal and tissue testing models with approaches that simultaneously assess large numbers of compounds over a wide chemical space with reduced cost per substance.

Chemical prioritization efforts and structure activity prediction modeling often utilize activity calls as input (e.g., Johnson et al. 2009; Martin et al. 2011; Reif et al. 2010) and, consequently, depend on consistent and reliable methods for making activity calls from the underlying data. However, the incomplete concentration–response profiles frequently observed in qHTS data render nonlinear statistical modeling and parameter testing challenging. It is not possible to determine whether response variances are homoscedastic (constant) or heteroscedastic (not constant) in unreplicated data sets, and few degrees of freedom may be available for statistical testing after curve fitting and outlier detection. Furthermore, traditional methods to assess nonlinear regression model fits depend on graphical diagnostics, but visual inspection of residual plots is not feasible in the qHTS analysis context that can involve thousands of compounds and hundreds of assays. An approach to activity call evaluation was developed here in response to these concerns.

The proposed three-stage activity call algorithm accommodates large volumes of qHTS data and does not require replicate assessments. Actives and inconclusives must produce a response that exceeds the assay detection limit and meet a prespecified statistical significance threshold. However, while the *p*-values obtained from statistical testing are based on uncertainty in model fits, in practice the true errors are not known. Calculated error estimates may be too low (producing false positives) or too large (producing false negatives) in some instances. In this study, data were simulated under a variety of scenarios (Table 2) to quantify algorithm performance over a broad range of possible profiles. Nevertheless, when dealing with large chemical libraries it may be useful to employ moderated test statistics like those developed for DNA microarray analyses to stabilize variance components [e.g., Cui et al. (2005); Smyth (2004)].

Similar to a PTE approach (S. Peddada, personal communication), the method described here performs well under conditions of homoscedasticity and heteroscedasticity (Table 4). The algorithm also performs well for moderate-to-high response levels across a broad range of parameter space (Figure 3) and with as few as four data points when |*RMAX*| ≥ 50% and *AC_50_* ≤ 0.1 μM (Table 5). The method can identify substances with full concentration–response curves as well as compounds inducing activity below the lowest tested concentration. The procedure effectively distinguishes substances with robust concentration–response profiles (ACTIVE*[±1] and ACTIVE*[±2]) and compounds with nonrobust concentration–response profiles (INCONCLUSIVE*[±3]). Even so, inconclusive calls may correspond to real activity and can be considered active when there is increased concern to minimize false negatives (e.g., toxicity studies). Compounds without sufficient evidence for activity within the tested concentration range are placed into a final category (INACTIVE*).

The effects of sample size (*n*) are summarized in Table 5. A small *n* may result from study designs with < 14 data points, data discarded due to experimental failure, or outlier removal during curve fitting. Type I error rates were < 0.03 in every case examined here (σ = 25%), whereas power and performance varied across parameter configurations. The performance of the classification algorithm was good (AUC > 0.9 in most cases) for almost all examined sample sizes (*n* = 4, 7, 9, 11, 13, 14) with |*RMAX*| = 100% and *AC_50_* set to 0.001 μM or 0.1 μM. The algorithm performed well (AUC ≥ 0.75) under almost all scenarios in which the |*RMAX*| ≥ 25%, the modeled detection limit of the qHTS assay.

The three-stage algorithm can be implemented in two steps in the freely available statistical software R (R Development Core Team, Vienna, Austria). Step 1 generates NLS and WNLS curve fits. Step 2 generates activity calls and other summary statistics from the output of Step 1. A computer with an Intel® Xeon® E5430 processor (2.66 GHz) and 2.92 GB of RAM was used with the Microsoft Windows® XP Professional Service Pack 3 operating system to obtain execution times for 1, 10, 100, and 1,000 chemicals. Due to possible memory constraints, it is recommended to use Linux machines when analyzing more than a few thousand chemicals at a time. For Step 1, the run times were approximately (in seconds) 1.5, 6.2, 53.1, and 520.4, respectively, for NLS curve fits and 2.0, 8.2, 53.8, and 532.3, respectively, for WNLS curve fits. For Step 2, the run times to generate activity calls were approximately (in seconds) 0.1, 0.2, 1.9, and 18.5, respectively. The R code for the three-stage algorithm and all simulated data are available upon request.

## Conclusion

An automated approach was developed to reliably classify concentration–response data into toxicologically relevant categories: actives (ACTIVE*[±1] or ACTIVE*[±2]), inconclusives (INCONCLUSIVE*[±3]), and inactives (INACTIVE*). The algorithm strategically uses both unweighted and weighted statistical testing in a multiple-decision framework. Active substances are subdivided in two types: ACTIVE*[±1] compounds exhibit concentration–response curves within the tested concentration range, whereas ACTIVE*[±2] substances have already achieved maximal response (or nearly maximal response) at the lowest tested concentration. The approach performed better than single-stage testing approaches and provides insight into nonlinear modeling in high-throughput toxicology.

## Supplemental Material

(971 KB) PDF
