# Supplementary material for: A Three-Stage Algorithm to Make Toxicologically Relevant Activity Calls from Quantitative High Throughput Screening Data
Source: Environ Health Perspect. 2012 May 10;120(8):1107–15. doi: 10.1289/ehp.1104688 (PMC3440085; doi:10.1289/ehp.1104688)
Supplement: (971 KB) PDF [file ehp.1104688.s001.pdf]

Supplemental Material

A Three-Stage Algorithm to Make Toxicologically Relevant Activity Calls from Quantitative High Throughput Screening Data

Keith R. Shockley

Biostatistics Branch, National Institute of Environmental Health Sciences, National Institutes of Health, Research Triangle Park, NC 27709; Biomolecular Screening Branch, Division of the National Toxicology Program, Research Triangle Park, NC 27709

Table of Contents

Curve Fitting 3

Statistical Testing...3

Simulations 4

References..6

Supplemental Material, Table S1 7

Supplemental Material, Figure S1...8

Supplemental Material, Figure S2...9

Supplemental Material, Figure S3...10

Curve fitting. The function drm() in the R package “drc” (Ritz and Streibig) was used to fit curves one substance at a time using the “BFGS” non-linear constraint option and restricting results to positive *SLOPE* parameters. Outlier detection follows a two-step algorithm described previously (Wang et al. 2010). The sum of squared residuals (SSR) comparing the four-parameter Hill model to a null model (i.e., mean response) is computed for a grid of initial parameter values spanning the simulated curve regions. The parameter set corresponding to the smallest SSR is selected as initial conditions for each curve fit. The final set of parameter estimates is found using unweighted non-linear least squares (NLS) or weighted non-linear least squares (WNLS). In the case of ties, the model with the lowest AC_{50} value is selected as the “best” fit.

Statistical testing. An overall F-test compares the SSR of the best fit to the four-parameter Hill equation (see Equation [S1] below) to the SSR of a horizontal line (flat model) for each substance. Significance is obtained from the F distribution. The WNLS criterion weights each response point i from n concentrations (R_i, i=1,…,n) based on similarity between R_i and responses obtained from test concentrations within a specified distance d of the concentration C_i corresponding to R_i. The concentration range $[C_i-d, C_i+d]$ is defined for weighting, where d is based on a fold-change criterion (3-fold change is selected for the current study), so that more influence is given to data points exhibiting similar response levels within $[C_i-d, C_i+d]$. For x concentrations, the weighting scheme is $w_i = 1/s^2$, i=1, …, x, where s is the sample standard deviation estimated from all response data within $[C_i-d, C_i+d]$. The overall F-test for the WNLS approach is based on the weighted SSR.

For the weighted t-test, the mean and sample standard deviation from Student's t-statistic are replaced by the weighted mean $\frac{\sum w_i R_i}{\sum w_i}$ and weighted sample standard deviation

$$\sqrt{\frac{\sum w_i \sum \left(w_i \times \left\{ R_i - \frac{\sum w_i R_i}{\sum w_i} \right\}^2 \right)}{\left(\sum w_i\right)^2 - \sum w_i^2}}.$$

Simulations. The form of the Hill Equation model for response (R_{ijkl}) of a single substance is,

$$R_{ijkl} = R0 + \frac{RMAX_j - R0}{\left(1 + \left(\frac{2^{\log_2 AC50_k}}{2^{C_i}}\right)^{SLOPE_l}\right)} + \varepsilon_{ijkl} \qquad [S1]$$

where C_i denotes the ith concentration, $RMAX_j$ denotes the jth maximal response, $R0$ is the minimal response, $AC50_k$ represents the kth concentration of half maximal response, $SLOPE_l$ denotes the lth hill slope coefficient, and ε_{ijkl} is the substance specific residual error. The values of C_i are based on μM units from qHTS designs used to assess nuclear receptor activity within Phase I of Tox21 (see Supplemental Material, Table S1). Accordingly, C_i values are set to (4.90 x 10^{-4}, 2.45 x 10^{-3}, 1.23 x 10^{-2}, 2.74 x 10^{-2}, 6.13 x 10^{-2}, 1.38 x 10^{-1}, 3.07 x 10^{-1}, 6.85 x 10^{-1}, 1.53, 3.43, 7.66, 17.13, 38.31, 76.63).

Three cases were explored, where $R0$ was set to 0 for every simulated data set. In Case 1, the values of $|RMAX_j|$ and $AC50_k$ were set to (25, 50, 100) and (10^{-3}, 10^{-1}, 10), respectively, which span the range of concentrations and responses generally observed in qHTS data. The

$SLOPE_l$ parameter was set to 1. Residual errors, ε, were modeled as $\varepsilon_{ijkl} \sim N(0, \sigma_i^2)$ for σ_i = (5%, 10%, 25%, 50%, 100%, $f(C_i)$=9.7355 + 0.1146 x C_i) where σ_i is expressed as percent of positive control activity. The linear function $f(C_i)$ represents the case of linearly increasing error with increasing concentration and is based on the relationship of σ_i to concentration derived from data in a set of recently published nuclear receptor agonist assays (see Huang et al. 2011; Supplemental Material, Table S1). For contour plots, $|RMAX_j|$ was set to (10, 25, 50, 75, 100, 125, 150) and $AC50_k$ is set to (10^{-4}, 10^{-3}, 10^{-2}, 10^{-1}, 1, 10, 100). Case 2 assesses the effect of $SLOPE_l$ parameter, in which the values of $SLOPE_l$ were set to (10^{-2}, 10^{-1}, 0.5, 1, 2, 10, 100) and σ = 25% with the same values of $|RMAX_j|$ and $AC50_k$ that were used in Case 1. Case 3 examines six different scenarios at σ = 25% in which a defined number of randomly selected data points were removed to produce an effective sample size *n* (*n* = 4, 7, 9, 11, 13, 14). The same values of $|RMAX_j|$, $AC50_k$ and $SLOPE_l$ were used in Case 3 as defined above for Case 1.

References

Huang R, Xia M, Cho MH, Sakamuru S, Shinn P, Houck KA, Dix DJ, Judson RS, Witt KL, Kavlock RJ, Tice RR, Austin CP. 2011. Chemical genomics profiling of environmental chemical modulation of human nuclear receptors. Environ Health Perspect 119:1142-1148.

Ritz C, Streibig JC. 2005. Bioassay analysis using R. J Stat Softw 12:1-22.

Venables WN, Ripley BD. 2002. Modern applied statistics with S. Fourth Edition. New York:Springer.

Wang Y, Jadhav A, Southal N, Huang R, Nguyen DT. 2010. A grid algorithm for high throughput fitting of dose-response curve data. Curr Chem Genomics 4:57-66.

Supplemental Material, Table S1. Calculated sample standard deviations of response data from nuclear receptor agonist assays[a]

	Assay									
**Concentration (μM)**	**AR**	**ER**	**FXR**	**GR**	**LXR**	**PPAR**	**PPARgamma**	**RXR**	**TRB**	**VDR**
4.90×10^{-4}	7.5	5.5	2.3	6.3	6.3	7.6	3.7	6.7	15.8	4.5
2.45×10^{-3}	8.1	6.9	2.4	5.3	6.4	3.8	2.3	10.3	13.8	1.9
1.23×10^{-2}	10.9	11.3	2.2	4.9	7.1	10.5	2.3	5.8	13.6	2.2
2.74×10^{-2}	9.7	12.0	2.9	5.5	8.2	5.2	2.3	7.3	12.3	2.7
6.13×10^{-2}	11.3	9.4	3.6	6.0	8.3	7.2	2.5	8.8	16.4	2.3
1.37×10^{-1}	14.1	6.5	2.6	5.5	6.2	4.8	2.5	7.0	13.9	2.1
3.07×10^{-1}	14.2	11.4	2.6	6.8	6.5	8.3	3.0	7.4	14.7	2.1
6.85×10^{-1}	11.6	8.9	4.0	5.9	7.0	5.1	2.8	6.7	14.0	2.2
1.53	12.9	8.8	2.5	5.9	8.4	8.6	3.4	6.8	14.7	2.4
3.43	13.4	10.3	3.5	6.2	9.0	11.3	4.2	8.6	14.1	5.1
7.66	16.5	22.2	4.7	7.9	10.8	11.4	5.8	11.0	14.5	5.3
17.1	17.3	15.6	5.5	8.0	13.2	12.1	7.1	15.9	14.8	5.7
38.3	18.9	FAILED[b]	6.3	7.6	16.4	13.3	8.7	16.4	17.1	6.0
76.6	30.4	18.4	8.0	9.1	FAILED[b]	17.4	9.4	17.2	23.4	11.3

[a]Expressed as percentage of positive control activity, see Huang et al (2011).
[b]Failed assay.

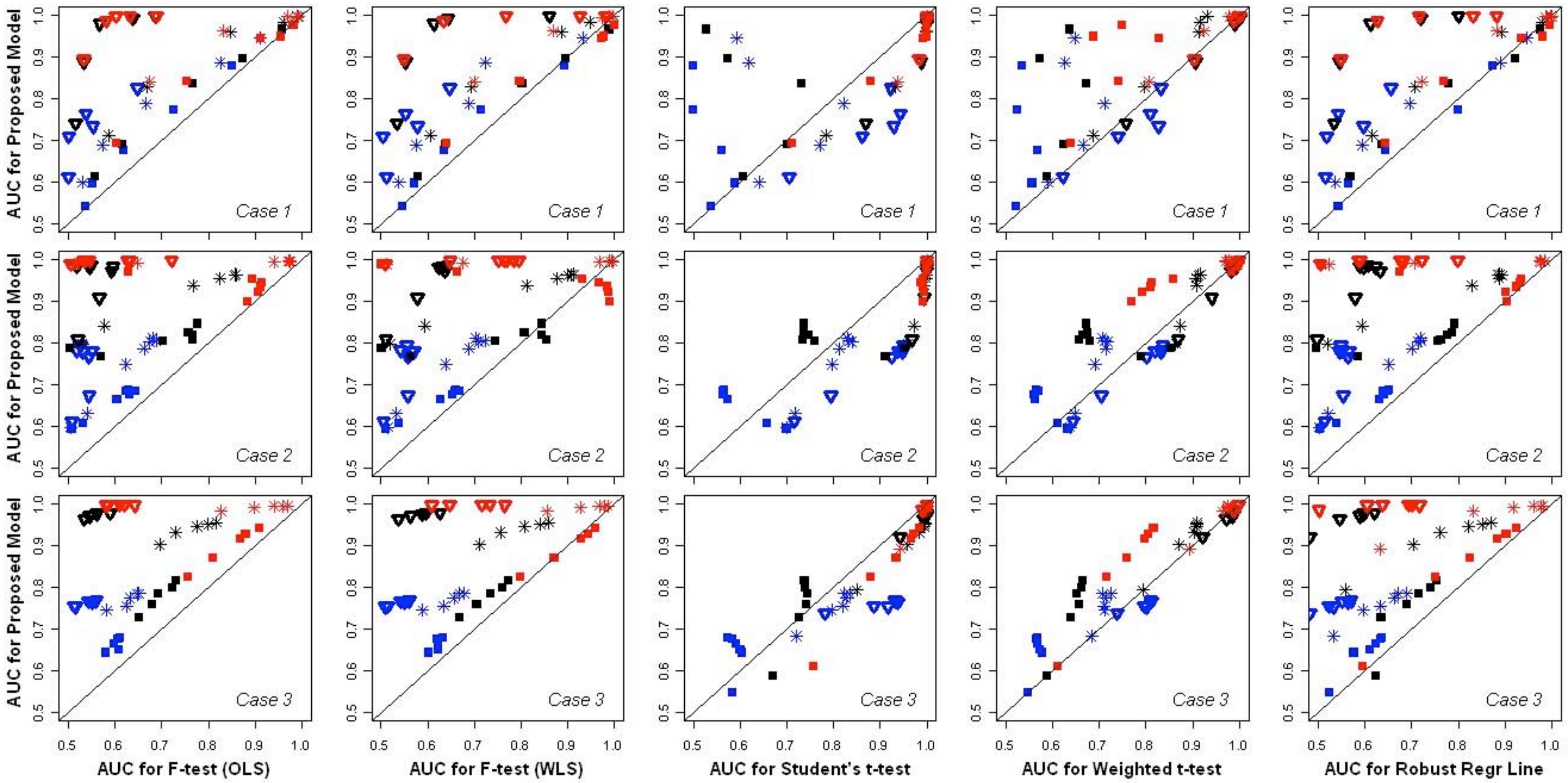

Supplemental Material, Figure S1. Area under the curve (AUC) of receiver operating characteristic (ROC) curves generated from simulated data sets with different error structures (Case 1), different slope parameters for σ = 25% (Case 2) and different sample sizes for σ = 25% (Case 3). AUC values are plotted for the proposed model versus activity calls obtained from two versions of the overall F-test (NLS and WNLS approaches described in the Supplemental Material, pages 3-4), two versions of the t-test (Student's t-test obtained from t.test() in R and a weighted t-test approach described in the Supplemental Material, pages 3-4) and a robust regression line obtained using the rlm() function in the R package "MASS" (Venables and Ripley 2002). Points colored blue refer to situations in which |*RMAX*| = 25% of the positive control, black points refer to |*RMAX*| = 50% of the positive control and red points refer to |*RMAX*| = 100% of the positive control activity. Triangles indicate *AC50* = 0.001 µM, asterisks indicate *AC50* = 0.1 µM and solid squares refer to *AC50* = 10 µM. The significance level for statistical testing is 0.05.

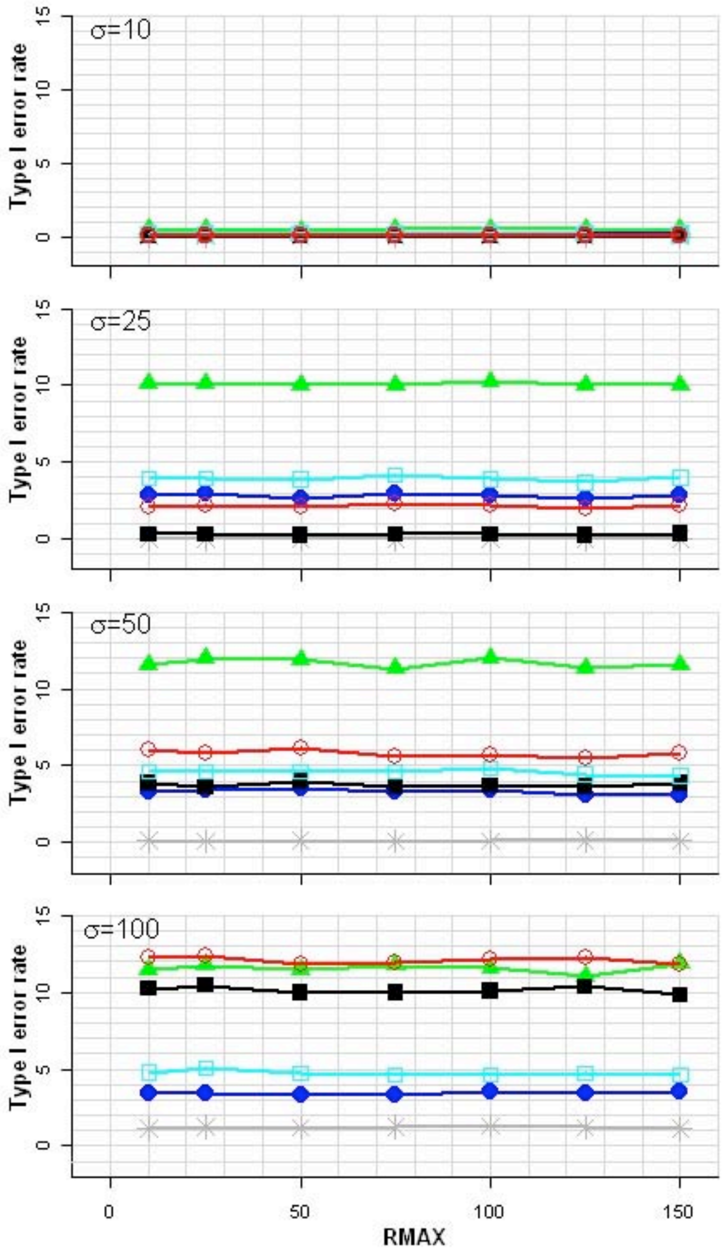

Supplemental Material, Figure S2. Type I error rates for different residual error structures. The Type I error rates are shown for the proposed model (red), the overall F-test using NLS (blue) and WNLS (green) described in the Supplemental Material, pages 3-4, Student's t-test calculated using the t.test() function in R (gray), a weighted t-test (black) described in the Supplemental Material, pages 3-4, and a robust regression line (cyan) calculated using the rlm() function from the R package "MASS" (Venables and Ripley 2002). The significance level for statistical testing is 0.05.

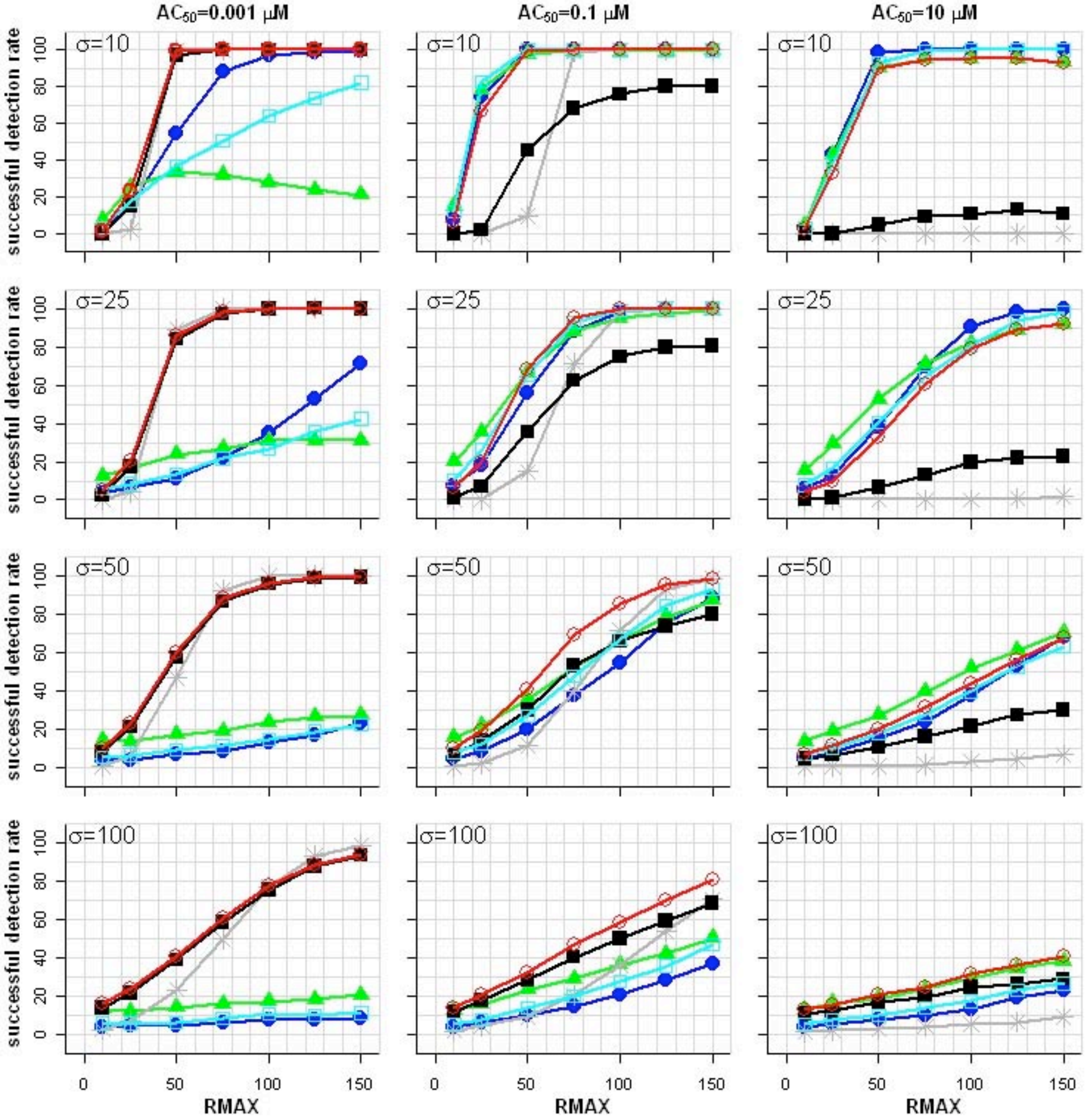

Supplemental Material, Figure S3. Statistical power for different residual error structures. The successful detection rates of true actives are shown for the proposed model (red), the overall F-test using NLS (blue) and WNLS (green) described in the Supplemental Material, pages 3-4, Student's t-test calculated using the t.test() function in R (gray), a weighted t-test (black) described in the Supplemental Material, pages 3-4, and a robust regression line (cyan) calculated from the rlm() function in the R package "MASS" (Venables and Ripley 2002). The significance level for statistical testing is 0.05.
